# Anti-Viral Drugs for Human Adenoviruses

**DOI:** 10.3390/ph3103343

**Published:** 2010-10-25

**Authors:** Mary Miu Yee Waye, Chor Wing Sing

**Affiliations:** School of Biomedical Sciences, Croucher Laboratory for Human Genomics, The Chinese University of Hong Kong, Shatin, N.T., Hong Kong

**Keywords:** adenovirus, pharmaceuticals, microRNA

## Abstract

There are many stages in the development of a new drug for viral infection and such processes are even further complicated for adenovirus by the fact that there are at least 51 serotypes, forming six distinct groups (A–F), with different degree of infectivity. This review attempts to address the importance of developing pharmaceuticals for adenovirus and also review recent development in drug discovery for adenovirus, including newer strategies such as microRNA approaches. Different drug screening strategies will also be discussed.

## Introduction

Human adenoviruses have been implicated as infectious agents which are responsible for numerous diseases, including respiratory tract infections, ocular and gastrointestinal tract disorders [1]. Adenoviruses usually cause mild, self-limiting respiratory illnesses, primarily in children, due to normal host responses which include the natural innate immune response involving the induction of cytokines and activation of effector leukocytes [2]; however, potentially fatal disseminated disease in highly immuno-compromised patients have been reported, particularly pediatric bone marrow transplant recipients. The treatment of such viral infections is not straightforward as with most viral infections and anti-viral research is faced with challenges such as highly divergent strain differences causing similar diseases. Currently, no Food and Drug Administration (FDA) approved treatment protocol has been used for treating adenovirus infection, nor are there any prospective randomized controlled trials of potentially useful anti-adenovirus therapies, though some antiviral drugs have been used in certain patients, e.g. cidofovir is used as a broad spectrum anti-viral agent [3,4,5,6] or donor leukocyte infusion as a therapy of life-threatening adenovirus infections after T-cell-depleted bone marrow transplantation [7].

## Adenovirus Biology and Structure

**Figure 1:**
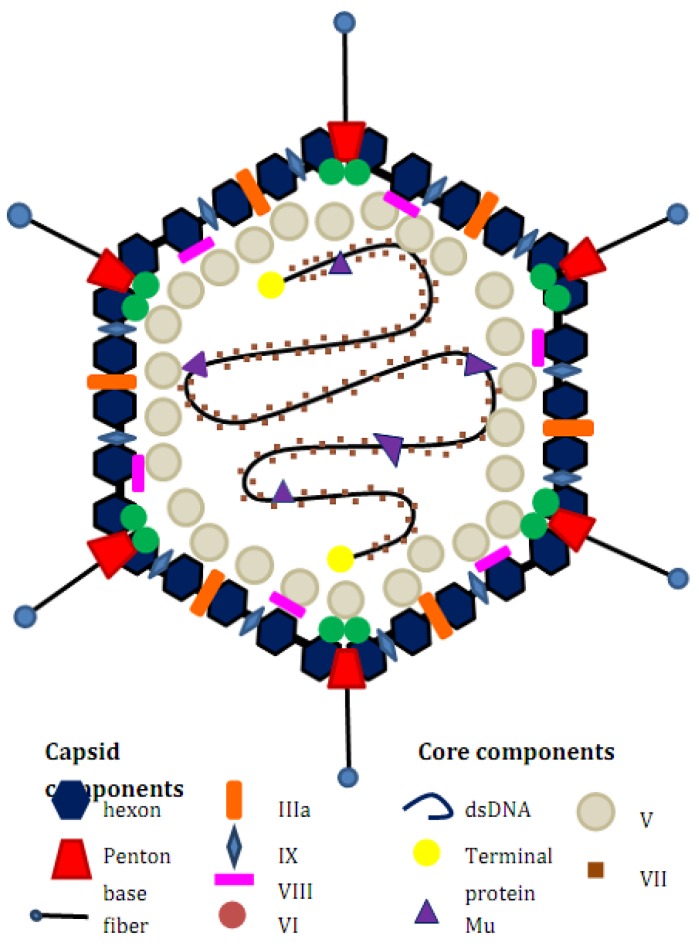
Structure of an adenovirus.

An adenovirus is a non-enveloped, dsDNA virus. There are at least 51 human Adenovirus serotypes which are classified into six groups (A to F) based on their biochemical, immunological and morphological criteria. Adenoviruses are about 90 to 100 nm in size and their structure consists of an icosahedral capsid which is made up of three major components: hexon located on the faces and edges of capsid; penton base located on the 12 fivefold apices; thin fibres attached on the penton base. (Figure 1). Other minor components: IIIa, VI, VIII and IX are also associated with the capsid. Inside the capsid are the virion protease which plays a vital role in the assembly of the virion and the double-strand DNA genome associated with five polypeptides (terminal protein (TP), V, VII, Mu and IVa2).

Infection by adenovirus starts with the binding of fibers to a specific receptor on the cell surface. The penton base interacts with the surface protein *av* integrins which stimulate the entry of the virus into the cell via endocyctosis. Acidification of endosome releases the virion into the cytoplasm and the virion migrates to the nuclear. Inside the nuclear, Adenovirus particles disassemble to release viral DNA for replication of the virus. 

**Figure 2:**
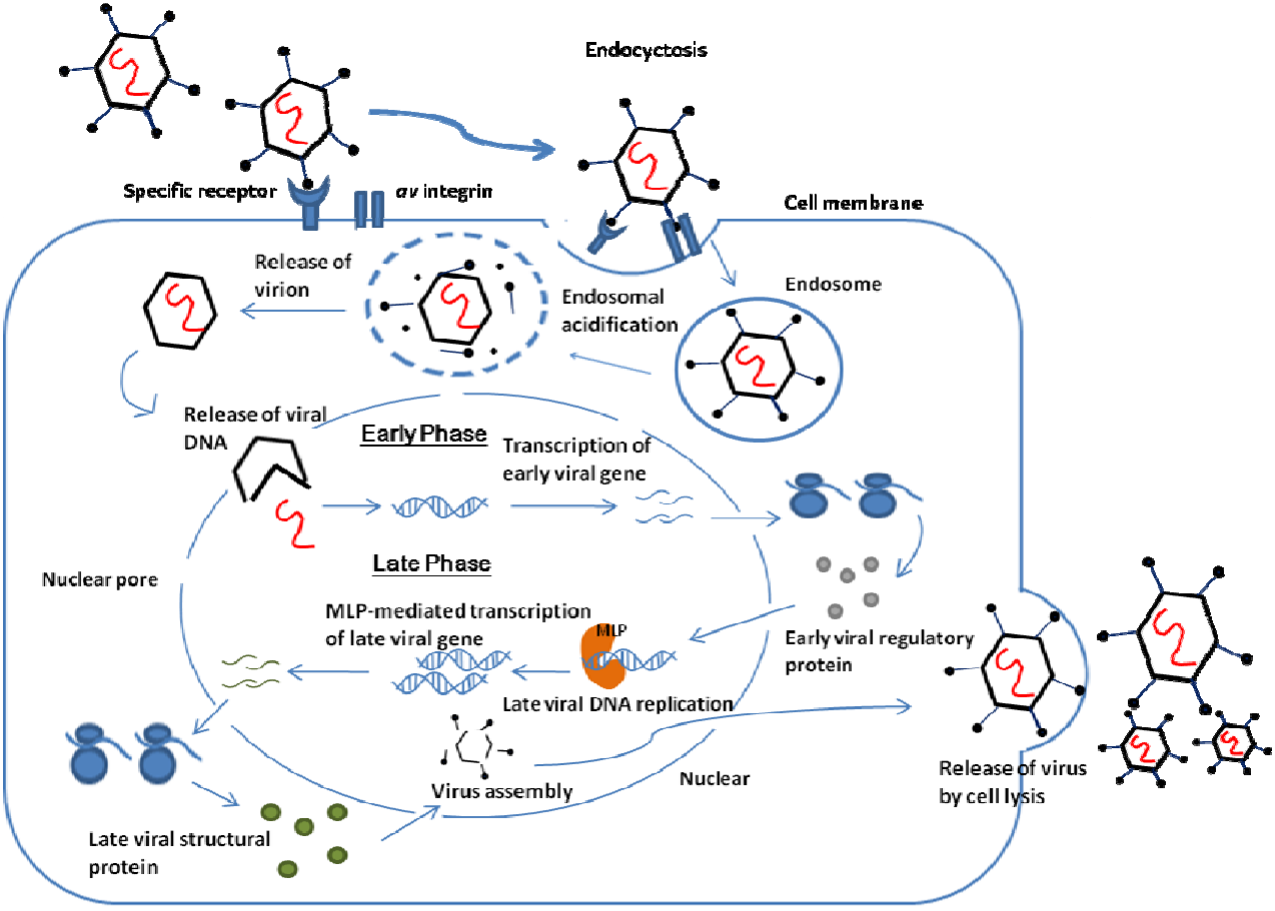
Adenovirus infection and replication pathway.

The adenovirus replication cycle is divided into two stages: early phase and late phase. The early phase expresses regulatory proteins which function to activate other virus genes, to avoid premature death of infected cells, and to alter the expression of host proteins for DNA synthesis. Once the components for DNA replication are ready, the late phase can start. The strong major late promoter (MLP) mediates the transcription of late virus genes which encode the viral structural proteins and proteins for the maturation of viral particles. The virus is assembled into its virion and released to infect other cells via virally induced cell lysis. Figure 2 summarized the infection cycle of adenovirus. and details of the replication of adenovirus can be found in the review of Russell [8].

## Rationale for Treatment

Adenovirus infection is commonly found in immunocompetent persons. Depending on the infecting serotypes, the infection can cause various illnesses such as respiratory tract disease, gastroenteritis, conjunctivitis and haemorrhagic cystitis. The symptoms of these illnesses are generally mild and can mostly be cured. However, in rare cases, severe diseases such as hepatitis, myocarditis and nephritis can be observed [9]. If ocular infection occur, adenovirus may even cause epidemic keratoconjunctivitis (EKC), resulting in decreased visual acuity [10].

In immunocompromised patients such as haematopoietic stem cell transplants (HSCT) recipients and HIV patients, adenovirus infection may easily result in death. It is found that, in retrospective studies of transplant patients, bone marrow transplant recipients have a high risk of adenovirus disease, especially with the use of alemtuzumab for pediatric patients receiving stem cell transplants from alternate donors [11,12,13]. Patients with graft-versus-host disease (GvHD), T-cell depletion of the graft and receipt of an unrelated donor transplant are also at a high risk of adenovirus infection [14]. In addition, the risk for pediatric patients is three times higher than that for adult patients [15]. 

Apart from fatal infections, adenovirus may also interfere with drug treatment and gene therapy. It has been shown that infections and normal flora in nonhuman primates have a negative impact on the efficacy of drug development [16]. Similarly one can imagine that in humans, adenovirus can also have an impact on drug administration: namely dosage and treatment protocols. For gene therapies that involve using adenovirus as a vector, pre-existing infection could lead to lower efficacy and sometimes severe immune system reaction could lead to multiple-organ-system failure [17]. In cystic fibrosis patients who receive gene therapy involving adenovirus as vectors, numerous studies have been done to neutralize antibodies against adenovirus so as to reduce the impact of pre-existing adenovirus [18].

Recently, Adenovirus 36 has been linked with human obesity. A survey from three different states in the United States (New York City, Madison, WI, and Naples, FL) showed a higher prevalence of Adenovirus-36 antibodies in obese compared to non-obese subjects [19]. In the whole population, antibody-positive individuals were 9 BMI units heavier than antibody-negative individuals [20]. These findings are supported by results from twin pairs discordant for Adenovirus-36 antibodies where antibody-positive twins were slightly, but significantly, heavier and fatter than their antibody-negative co-twins [20]. Thus it is conceivable that metabolic syndromes caused by obesity could be treated by anti-adenovirus therapy. 

## Current Anti-Viral Drugs

Though adenoviruses have been identified for years, there is no FDA approved drug for human adenovirus infection. Currently, only two antiviral drugs, namely cidofovir and ribavirin, are being used in first-line adenovirus therapy. 

### Cidofovir

Cidofovir [(*S*)-HPMPC; (*S*)-1-(3-hydroxy-2-phosphonylmethoxypropyl)cytosine)] is an acyclic nucleoside phosphonate which shows anti-viral activity against DNA viruses [21]. It inhibits the ADV replication, independent of the serotype, by acting as a monophosphate form of nucleotide. With phosphylations by cellular kinases, the compounds are activated to become 2’deoxyribonucleoside 5’- triphosphates (dNTPs) analogues. Viral DNA polymerases bind to the analogues, resulting in the interference with the viral replication process. This targeting action of cidofovir is validated by isolating the cidofovir-resistant adenovirus mutants which contain distinct sequences near the conserved nucleotide binding site of adenovirus DNA polymerases [22].

### Ribavirin

Ribavirin is a broad spectrum antiviral agent showing activity towards adenovirus infections. Like cidofovir, ribavirin is a nucleoside analogue but whether its target of action is same as viral DNA polymerases is still controversial. Other suggested mechanisms include the direct inhibition of adenovirus infection by immunomodulation or depletion of intracellular guanosine triphosphate pools and indirect inhibition by interference with RNA capping or induction of mutation [23]. Although ribavirin is a broad spectrum antiviral agent, *in vitro* study in HSCT patients found out that the efficacy of ribavirin is restricted to species C adenovirus infection only [24].

## Efficacy as Established in Clinical Studies

### Cidofovir

Cidofovir has completed clinical trials and is approved as a drug to treat CMV retinitis in AIDS patients. A number of clinical studies have already shown the efficacy of cidofovir to the treatment of acute adenoviral keratoconjunctivitis [25,26]. In addition to ocular adenovirus infection, retrospective studies in immunocomprised patients also showed efficacy against adenovirus infection [27,28,29]. When combined with other anti-adenovirus therapies e.g. IVIG therapy, more promising result was obtained [30], but the individual efficacy of cidofovir is difficult to estimate. In most of the studies, efficacy was higher when earlier treatment was applied after diagnosis of adenovirus infection [31]. Although cidofovir is an effective anti-adenovirus drug, a severe CNS side effects and retinal toxicity have been observed after cidofovir administration [32].

### Ribavirin

While cidofovir showed efficacy against adenovirus infection, conflicting results were observed for ribavirin treatment in adenovirus infected patients. In some case reports, ribavirin successfully treated the immunocomprised patients with adenovirus infection [33,34,35]. However, when a larger-scale studies were carried out, no significant efficacy was observed [36]. This conflicting result can be explained by the selective efficacy in adenovirus serotypes. Compared to cidofovir, ribavirin has less toxicity and side effects in the patients.

## Antiviral Drugs Currently in Clinical Trials

### CMX001

CMX001 (Chimerix, 3-hexadecyloxy-1-propanol-cidofovir), is a lipid conjugate of cidofovir which is being developed as an antiviral drug. It shows antiviral activity against all double-strand DNA viruses, including adenovirus 3, 5, 7, 8, 31 [37]. This drug is orally administrated and is designed to cross the intestinal wall and penetrate into the target cell before being cleaved to release cidofovir for its action. Result from phase I clinical Trial has illustrated the CMX001 oral bioavailability in humans and has demonstrated a positive safety profile (ClinicalTrials.gov Identifier: NCT00780182). Another study showed that CMX001 has a higher antiviral activity than cidofovir with less toxicity to patients [38]. Now the drug is being studied in Phase II which investigates its efficacy in immunocomprised patients with CMV infection (ClinicalTrials.gov Identifier: NCT00942305). 

## Potential Anti-Viral Drugs

Many studies illustrate that varies compounds and molecules have the anti-adenovirus activity, which have the potential to be developed into potent drugs against adenovirus.

### DHEA and epiandrosterone (EA) analogue

DHEA, EA and two synthetic derivatives present anti-adenovirus activity similar to cidofovir. A study showed that these steroidal compounds inhibit adenovirus protein synthesis and replication with selectivity indices ranging between 42 and 83 [39].

### Transition metal complexes

[Co(NH_3_)_6_]Cl_3_, a transition metal complex, is a broad-spectrum anti-viral compound against Sindbis virus. Due to its high positive charge density, the metal complex binds strongly to the negatively-charged nucleotides and causes condensation of viral dsDNA into toroidal-like superstructure, disturbing the viral DNA packaging process. A study by Knight *et al*. showed that with the presence of [Co(NH_3_)_6_]Cl_3_, cells infected with adenovirus survived better and the viral expression of infected cells decreased, demonstrating that [Co(NH_3_)_6_]Cl_3_ has anti-viral activity towards adenovirus [40].

### Bispecific monoclonal antibodies

The 2-armed structure of MAb allows researchers to place a therapeutic agent on one arm while the other arm specifically targets the disease site. This unique structure holds great promise for numerous therapeutic needs including adenovirus infection. With the use of MAb, adenoviral gene therapy may decrease the toxicity by lowering the dose of adenovirus used [41].

### Camptothecin (CPT)

The anticancer drug camptothecin (CPT) have been shown to be a potent inhibitor of replication, transcription and packaging of double-stranded DNA-containing adenoviruses. It binds to topoisomerase II, a host cell enzyme required for initiation and completion of the viral functions, and thus avoids the replication of the virus [42].

### Water-soluble polymer complex of Arbidol

Arbidol is a domestic antiviral agent with broad-spectrum antiviral activity. It inhibits the fusion of virus lipid shell with membrane of endosomes located within the cell. Due to water insoluble property, arbidol has a low bioavailability and high toxicity. To overcome these limitations, water-soluble complexes have been synthesized between arbidol and polymer compounds. Study showed that this complex has broad anti-viral activity including adenovirus while the toxicity is much lower than the non-modified arbidol. Thus, the complex can be useful in developing safe anti- adenovirus drugs [43].

### Stavudine

Stavudine is a dual-function anti-human immunodeficiency virus (HIV) agent. It acts as an analogue of thymidine and is phosphylated by cellular kinase to become stavudine triphosphate which terminates DNA synthesis by incorporating into it. Recent studies showed that stavudine selectively inhibited human AdV5, indicating a potent and selective anti-adenovirus activity. The lead compound of stavudine, stampidine, was found to be the most potent non toxic antiviral agent and it had a remarkable *in vitro* and/or *in vivo* efficacy against drug-sensitive and drug-resistant adenovirus. It is suggested that stampidine has clinical potential as a dual-function topical agent for the prevention and/or effective treatment of adenovirus [44]. 

### MicroRNA

Recent developments in our basic knowledge about microRNA have been applied in drug development. One such strategy is to clone the binding sites for certain microRNAs to decrease the expression of viral genes. A study by Cawood *et al*. showed that animals administered a ten-fold lethal dose of wild-type Ad5 (5×1010 viral particles/mouse) showed substantial hepatic genome replication and extensive liver pathology. However, if 4 microRNA binding sites were added, the genome replication decreased 50-fold and liver toxicity was virtually abrogated. MicroRNA should provide a new strategy for designing safe attenuated vaccines applied across a broad range of viral diseases [45].

### Intravenous Immunoglobulin (IVIG) therapy and T- cell immunotherapy

IVIG has also been used for adenovirus pneumonia in children, although the therapy controls but does not eradicate adenovirus unless stem cell immunotherapy is also given [46,47]. Although routine use of IVIG is common practice for indications such as allogeneic bone marrow transplant and primary immunodeficiencies, care should be exercised during administration of IVIG as adverse effects have been reported in 5–15 % of patients receiving IVIG and IVIG's effects usually last between 2 weeks and 3 months [48]. More recently, a successful case of combined use of CDV and IVIG in renal transplant patients with disseminated adenovirus infection has been reported [49].

## Current Drug Screening Methods

Drug screening is essential for the discovery of antiviral compounds. For human adenovirus, diverse *in vitro* antiviral assays exist and most are cell-based including cytopathic effect assay (measurement of plaque reduction) and MTT assay (measurement of cell variability). Other assays, such as ELISA, are also frequently used to detect the presence of adenovirus protein for cytotoxicity study of the drug. These antiviral assays are not standardized and time-consuming and therefore, other new methods are increasingly used for drug screening. 

## New Methods for Drug Screening

### RT-PCR method

More recently real time PCR-based antiviral assay have been used and were shown to be a more rapid and effective drug-screening test [50]. Some caution should be taken since in other assays with RT-PCR, it can be shown that 23 common respiratory pathogens have cross-reactions in certain assays (with intermittent cross-reaction to adenovirus at a high dosage of >10(7) TCID50/mL) [51]. With the use of real time PCR, the antiviral assay becomes rapid, reproducible and could replace classical and more labor-intensive infectivity assays.

### Biosensor Method using Capacitance Sensor Arrays

The capacitance sensor array could be a new method for antiviral drug screening. This array is used to detect adenovirus entry via receptor-mediated endocytosis, which is also an essential process for therapeutic gene/drug delivery that is targeted to a specific cell type. By screening which compounds act on the adenovirus targeting cell type, new antiviral drugs could be discovered [52].

### Computation Method

Bioinformatics and computational methods have been used to discover novel pharmaceuticals. With the bioinformatics tools and software, one can simulate drug-receptor interactions, predict drug bioavailability and bioactivity and illustrate the functional structure of the drug. Computational methods can be applied in antiviral drug screening and recently, p16(INK4a) peptide mimetics, which inhibit viral cell cycles, have been identified *via* virtual screening [53]. 

### Animal Model

The discovery of anti-adenoviral drugs has been hampered by the lack of a permissive immunecompetent model for *in vivo* study. However, recent research has identified a promising animal model for anti-adenoviral drug analysis [54]. Study on CMX001 showed that human adenovirus 5 infection in immunosupressed Syrian hamsters caused severe systemic diseases similar to that in immunocomprised patients [37]. This animal model allows replication of adenovirus and thus can be used to test the efficacy of anti-adenoviral drugs.

## Conclusions

In conclusion, there is a much need for the development of novel anti-adenovirus agents, including the prevention of fatal diseases from immuno-compromised patients, facilitating gene therapy trials that use adenovirus as a vector and for future development of anti-obesity schemes for those whose obesity might be linked to adenovirus 36. Potential anti-adenoviral drugs include chemicals that have broad-spectrum anti-viral activities, plus some newer approaches such as miroRNA strategies. These new drugs could be developed further with the help of advanced drug screening methods such as bioinformatics and computational methods, biosensors, molecular probes for PCR, together with the newly developed animal model. 
